# Can Follow-Up Examination of Tuberculosis Patients Be Simplified? A Study in Chhattisgarh, India

**DOI:** 10.1371/journal.pone.0051038

**Published:** 2012-12-05

**Authors:** Debashish Kundu, Ajay M. V. Kumar, Srinath Satyanarayana, Puneet Kumar Dewan, Sreenivas Achuthan Nair, Kshitij Khaparde, Priyakanta Nayak, Rafael Van den Bergh, Marcel Manzi, Donald A. Enarson, Madhav Rao Deshpande, Sachin Chandraker

**Affiliations:** 1 Office of the World Health Organization (WHO) Representative in India, WHO Country Office, New Delhi, India; 2 International Union against Tuberculosis and Lung Diseases (The Union), South East Asia Office, New Delhi, India and Paris, France; 3 Médecins Sans Frontières, Operational Centre Brussels (OCB), Luxembourg, Luxembourg; 4 Directorate of Health Services, Raipur, Chhattisgarh, India; 5 Intermediate Reference Laboratory, Raipur, Chhattisgarh, India; Universidade Federal do Acre (Federal University of Acre), Brazil

## Abstract

**Background:**

Each follow-up during the course of tuberculosis treatment currently requires two sputum examinations. However, the incremental yield of the second sputum sample during follow-up of different types of tuberculosis patients has never been determined precisely.

**Objectives:**

To assess the incremental yield of the second sputum sample in the follow-up of tuberculosis patients under the Revised National Tuberculosis Control Programme (RNTCP) in Chhattisgarh, India.

**Methodology:**

A record review of tuberculosis (TB) patients registered in 2009 using a structured proforma from two sources, Tuberculosis and Laboratory Register, was undertaken in the six districts of Chhattisgarh, India.

**Results:**

In smear positive cases, of 10,048 follow-up examinations, 45 (0.5%) were found to be smear positive only on the second sputum when the result of the first sample was negative. In smear negative pulmonary and extra pulmonary TB patients, of 6,206 follow-up smear examinations, 11(0.2%) were found to be smear positive.

**Conclusions:**

The incremental yield of a second smear examination was very low, indicating that examination of one sputum sample is enough during follow-up among TB patients. There is insufficient yield to support sputum smear microscopy for monitoring smear negative pulmonary TB and extra pulmonary TB patients. These results indicate that the follow-up smear microscopy can be substantially simplified with favourable resource implications.

## Introduction

In India, where nearly 1.5 million tuberculosis (TB) patients a year are reported by the Revised National Tuberculosis Control Programme (RNTCP), each patient undergoes two sputum smear examinations as follow-up on at least three occasions while on treatment to assess response to therapy [1].

Previous studies on the necessity of examining a second specimen on each follow-up occasion have given inconsistent results [2], [3]. However, these studies were localized and did not provide disaggregated information on the incremental yield of the second specimen for different types of TB (new and re-treatment TB, pulmonary and extra-pulmonary TB, sputum smear positive or negative) and at different periods during the course of TB treatment (at the end of intensive Phase (IP), continuation Phase (CP) and end of TB treatment). Consequently, it has not been possible to agree on a change in policy.

This study, undertaken in the state of Chhattisgarh, India aimed to assess the incremental yield of a second sputum sample for follow-up of different types of TB and at different occasions during the course of TB treatment following the existing RNTCP guidelines where the spot sample collected was the first sample and the early morning sample was the second sample, providing the detailed evaluation needed to determine whether a change in policy is justified.

## Methods

### Study Design

A cross sectional study using record review.

### Setting

Under the Revised National Tuberculosis Control Programme (RNTCP) in India, sputum smear examination forms the cornerstone of both diagnosis as well as monitoring response to treatment. All TB patients during the course of their treatment are expected to undergo follow-up sputum smear examination for acid-fast bacilli (AFB) by Ziehl-Neelsen staining at defined occasions (at least three times) based on their treatment category [1]. These occasions are; first, at the end of intensive phase (IP), second, two months into the continuation phase and the third, at the end of TB treatment. In addition, if the patient is smear positive at the end of IP, the IP is extended by a month and the patient undergoes sputum examination at the end of extended intensive phase [Table 1].

**Table 1:** Follow-up schedule for sputum collection and smear examinations for sputum positive TB patients.

	End of IP			Extended IP			2 months into CP			End of Treatment	
Category	Give sputum cup	Collect sputum*	Result by	Give sputum cup	Collect sputum#	Result by	Give sputum cup	Collect sputum#	Result by	Give sputum cup	Collect the sputumand result by end ofthe treatment
**New** **	22	23	24	34	35	36	8	9	10	17	18
**Previously Treated**	34	35	36	46	47	48	8	9	10	21	22

Two sputum samples are to be collected, one as early morning and the other as spot sample. (Source: Revised National Tuberculosis Control Programme’s Revised Module 1–4, April 2011).

* The intensive Phase is extended by four weeks (12 doses) in initially smear positive PTB patients who continue to be positive at the end of the 2 months of IP.

# Early morning and spot specimens will be collected on this day.

** The numbers during the IP represent doses of anti-TB drugs, wheras during the CP they represent weekly blister packs of anti-TB drugs.

Note: For “New” patients, the intensive phase lasts for two months (8 weeks, 24 doses) and is followed by the continuation phase of four months (18 weekly blister packs, 54 doses). For “Previously Treated” patients, the intensive phase consists of two months (8 weeks, 24 doses), followed by one month (4 weeks, 12 doses) and then the continuation phase of five months (22 weekly blister packs,66 doses). The doses are given thrice a week on alternate days.

On each occasion two sputum smears are examined; first a spot (taken directly from the patient at the time of the visit) and an early morning sputum specimen (collected by the patient at home, first thing in the morning). A patient is considered to be smear positive if either of the sputum samples is AFB positive.

### Study Site

The state of Chhattisgarh in central India (population 25 million) has 80% of the population living in rural areas and 30% considered ‘tribal’ (as notified by Government of India) with a history of extremism and conflict. Of 18 districts in the state, 12 have been classified as remote, tribal and extremist-affected areas.

The study was carried out in six districts selected by stratifying the state into three zones (North, Central and South Zones) and selecting the two best-performing districts in each zone defined by programme indicators, vis-à-vis, three months sputum conversion rate for all New Smear Positive tuberculosis patients of 90%; low percentage of High False Errors (HFE) and Low False Errors (LFE); availability of all key RNTCP and trained staff in the selected districts; and unique tribal population characteristics, comprising of two completely tribal districts and four partially tribal districts. These districts consisted of 22 tuberculosis units covering a total population of 8.5 million, each with a TB register of all patients enrolled on TB treatment under RNTCP in their designated geographical areas and 106 microscopy centers, each of which maintained a laboratory register. The formats of these registers were similar to that recommended by the World Health Organization (WHO). All six districts had functional External Quality Assurance (EQA) systems for sputum smear microscopy with satisfactory level of performance. During the study period across the six districts the percentage of HFE (0.25%) and LFE (0.18%) were low and percentage of QE (0.24%) was quite good, showing that all the six centers had functional EQA with nearly homogenous performance. The study was conducted between October 2011 and May 2012.

### Sample

Records of all pulmonary and extra-pulmonary patients registered for treatment under RNTCP in the calendar year 2009 in the six districts were included in this study.

### Variables, Data Collection and Analysis

Variables collected included name of district, tuberculosis unit, age (in years), sex, TB registration number, classification of TB (Pulmonary TB – Smear positive and Smear negative, Extra-pulmonary TB), type of TB (New or Re-Treatment – Failure, Treatment of Default, Relapse and Others), results of two follow-up smear examinations at end of intensive phase, extended intensive phase, mid continuation phase and end of continuation phase.

Data was collected by the programme staff, trained for the purpose by the Medical Consultants hired by the World Health Organization, using a ‘structured proforma’ from two sources: TB Treatment registers and Laboratory registers which were kept in the respective Tuberculosis Units and Designated Microscopic Laboratories. The trained programme staff then extracted the requisite information verbatim into the ‘structured proforma’ designed for the study purpose, minimizing the chance of data interpretation error. In situations, where there was a discrepancy between sputum smear results in TB register and that recorded in the Laboratory register, the results in the Laboratory register were considered final as Laboratory registers were the primary source of data for sputum smear results. The data was then entered in the Microsoft Excel by the respective data entry operators of the districts under the supervision of WHO-RNTCP Medical Consultants.

Records with missing, inconsistent and inaccurate information were excluded and the dataset was finalised and analysed using EpiData. Data were cross-tabulated and the following were calculated: the proportion of patients who were smear positive at each follow-up occasion, the proportion who were positive on the second sample only when the first sample was negative (ie., the “incremental yield” - X), and the its inverse (1/X) was determined to calculate the number of second sputum smear examinations, that were done to get one additional smear positive case.

### Ethics Approval

Since this study was a review of records and did not involve patient interaction, individual patient consent was deemed unnecessary. The protocol was reviewed and approved by the State TB Officer, Directorate of Health Services, Government of Chhattisgarh, Médecins Sans Frontières’ Ethics Review Board and the Ethics Advisory Group of International Union Against Tuberculosis and Lung Disease (The Union) with a waiver of individual patient consent.

## Results

A total of 10,128 TB patients were registered during the study period of whom, 785 (8%) were excluded because of missing values and inconsistencies in date of starting treatment and date of treatment outcome. Of the remaining, there were 3,976 new smear positive (NSP), 3,256 new smear negative (NSN), 1,300 new extra pulmonary (NEP), 315 treatment after relapse, 61 treatment after failure, 208 treatment after default (TAD) and 227 retreatment others (RTO) patients for whom the data were analyzed.

The results of the follow-up examinations, by type of smear positive TB and at different periods during the course of treatment (including incremental yield) are given in Table 2. Of 10,048 follow-up smear examinations, 288 (2.9%) were found to be smear positive on at least one of the specimens. On 45 (0.5%) occasions patients were found to be smear positive only on the second specimen when the result of the first sample was negative. The number of additional smear examinations required to detect one smear positive case on the second sample was 216 among smear positive TB patients.

**Table 2:** Smear-positive tuberculosis patients undergoing sputum smear examinations in monitoring anti-tuberculosis (TB) treatment in 2009 in six districts of Chhattisgarh State, India, by type of tuberculosis, yield and workload required to detect an additional case.

Type of TB	Number of follow-up examinations	Number smear positive		Additional cases detected on second smear after first smear is negative		Number of additional smears required to detect a smear positive case on second smear
	N	N	%	N	%	N
**New Smear Positive**						
End IP	3,643	174	4.8%	31	0.9%	112
Ext IP	140	12	8.6%	1	0.8%	128
Mid CP	1,786	15	0.8%	2	0.1%	871
End of Rx	3,375	16	0.5%	1	0.0%	3,345
Total	8944	217	2.43%	35	0.39%	256
**Re-treatment Smear Positive**						
End IP	469	31	6.6%	7	1.6%	63
Ext IP	26	9	34.6%	0	0.0%	NA
Mid CP	226	15	6.6%	1	0.5%	207
End of Rx	383	16	4.2%	2	0.5%	183
Total	1104	71	6.43%	10	0.91%	110
**Total follow-up examinations**	**10,048**	**288**	**2.87%**	**45**	**0.5%**	**216**

IP = Intensive Phase; CP = Continuation Phase; Rx = Treatment; Ext = Extended Intensive phase; N = Number.

NSN, NEP and RTO cases underwent follow-up smear examinations on 5052, 832 and 322 occasions respectively; only on 7 (0.1%), 0 (0%) and 4 (1.2%) occasions patients were found to be smear positive on at least one of the specimens respectively. Among the NSN, NEP and RTO, only 3 (0.05%) cases were detected as positive on the second sample when the result of the first sample was negative (not included in the table).

## Discussion

This is the first study to examine the incremental yield of the second sputum smear examination at various follow-up periods for different types of TB patients. The incremental yield of the second smear examination was very low in this large group of patients. Unlike the previous studies, we reviewed both the TB registers and laboratory registers from a large number of districts with a relatively well-functioning sputum smear quality assurance system. We believe that the data is accurate and representative of programme realities. The implications of the study results on policy and practice are as follows:

First, the incremental yield of the second sputum smear during the follow-up examinations was very low across all categories of TB patients including initially smear positive TB patients. Though the yield was relatively better among retreatment smear positive TB patients as compared to new TB patients, it was still very low. Hence, failing to carry out a second sputum smear examination will have negligible effect on monitoring response to therapy. Given that India is a resource-limited country and TB frequently occurs in socio-economically deprived populations, this will in fact benefit the patients by reducing the number of visits to health facilities as examination of second sample is usually done on an additional visit. The number of smears that need to be examined to find an additional positive on the second smear was about 216. Hence, the programme will also benefit from the savings in cost incurred for laboratory consumables and the precious time of the laboratory technicians, which can be used for further improving quality of smear microscopy. Of the 309,702 smears examined for smear microscopy in various microscopy centres of the state of Chattisgarh in 2011, 90,960 (29.6%) were examined during follow-up, and changing from two sputum examinations to one during follow-up can reduce the overall laboratory workload by 15%. Therefore, we strongly recommend discontinuation of examining a second sputum sample during follow-up of TB patients. Though already recommended by the International Union against Tuberculosis and Lung Disease [4] this policy has not been implemented widely.

Second, among sputum smear negative and extra pulmonary TB patients follow-up examinations were rarely found to be smear positive (0.2%). The yield is so low as to justify discontinuing this practice, supporting the 2009 WHO treatment guidelines [5].

The study had some limitations. First, we had no information on sputum smear grading which would have added additional value to our findings. Previous studies have indicated that distribution of smear quantification differs by the pattern of serial results of the two smears (both smears positive and only one smear positive) [2]. In follow-up examinations, where only one of the two smears was positive, most of them were graded scanty or 1+ indicating fewer bacilli and possibly non-viable [2]. Though, there are no studies which have examined if culture negativity is more likely in follow-up examinations which are positive only on single smear and if it varied with the smear quantification and can be a topic for future research. Second, we didn’t have information on drug susceptibility profile and HIV status of the TB patients as it was not routinely done under programmatic settings in 2009. The drug susceptibility profile can be different for re-treated and new patients and can also be a topic of future research to find out the influence of drug resistance on the incremental yield of the second sputum smear examination during follow-ups. Third, results of this study can be extrapolated only to the countries that still use only sputum smear microscopy during treatment to evaluate treatment response which is insensitive in comparison to sputum culture.

### Conclusions

The incremental yield of a second smear examination and its use in monitoring anti-TB treatment is negligible. There would be no major implications to the monitoring of TB patients’ response to therapy if the policy was changed from examination of two sputum smears to that of one during follow-up smear examinations and collect only one spot sputum sample for follow-up sputum examinations under proper guidance of the Laboratory Technician.
